# Supplementary material for: Regulation of nuclear transcription by mitochondrial RNA in endothelial cells
Source: eLife. 2024 Jan 22;13:e86204. doi: 10.7554/eLife.86204 (PMC10803041; doi:10.7554/eLife.86204)
Supplement: Figure 2—source data 1. — The expected PCR product formed in presence of RT (+) is indicated in solid red box and the gel image used for Figure 2B is indicated in dashed red box. [file elife-86204-fig2-data1.pptx]

## Slide 1
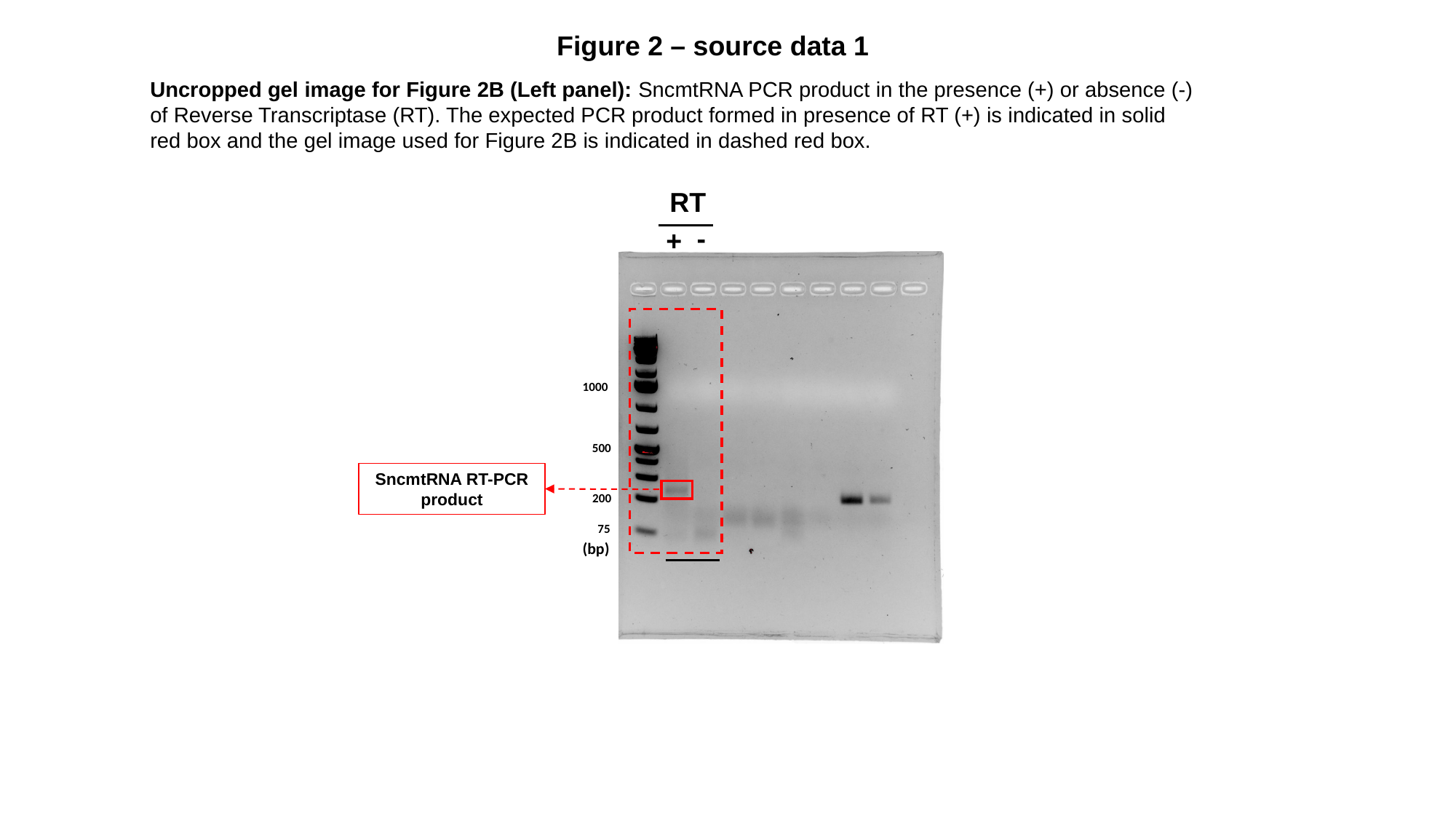

Figure 2 – source data 1
Uncropped gel image for Figure 2B (Left panel): SncmtRNA PCR product in the presence (+) or absence (-) of Reverse Transcriptase (RT). The expected PCR product formed in presence of RT (+) is indicated in solid red box and the gel image used for Figure 2B is indicated in dashed red box.
RT
-
+
1000
500
SncmtRNA RT-PCR product
200
75
(bp)
