# Supplementary material for: Regulation of nuclear transcription by mitochondrial RNA in endothelial cells
Source: eLife. 2024 Jan 22;13:e86204. doi: 10.7554/eLife.86204 (PMC10803041; doi:10.7554/eLife.86204)
Supplement: Figure 2—source data 2. — The PCR products formed for 16S rRNA, SncmtRNA, and β-actin are indicated in solid red box and gel image sections used for Figure 2B are indicated in dashed red box. [file elife-86204-fig2-data2.pptx]

## Slide 1
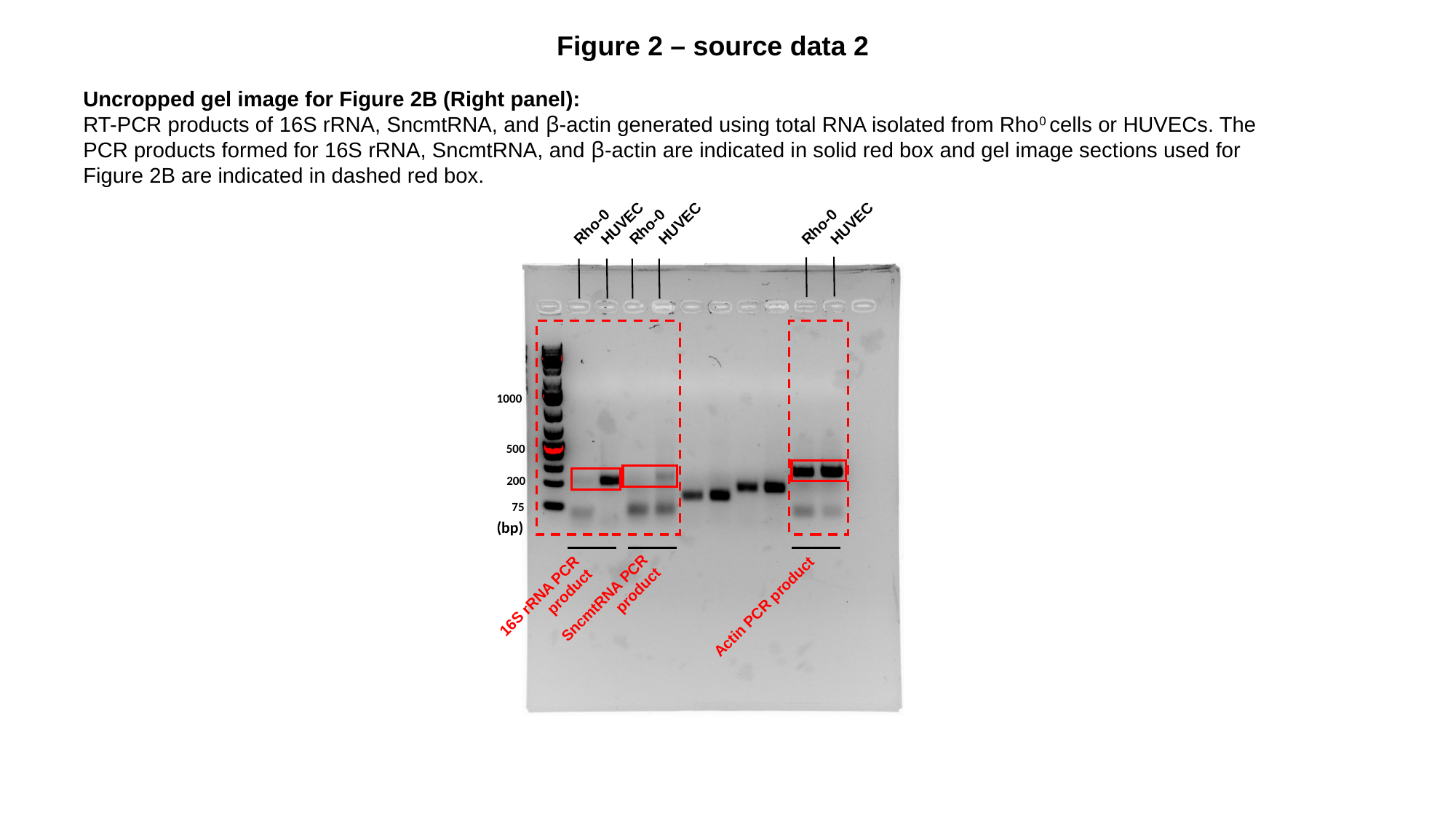

Figure 2 – source data 2
Uncropped gel image for Figure 2B (Right panel):
RT-PCR products of 16S rRNA, SncmtRNA, and β-actin generated using total RNA isolated from Rho0 cells or HUVECs. The PCR products formed for 16S rRNA, SncmtRNA, and β-actin are indicated in solid red box and gel image sections used for Figure 2B are indicated in dashed red box.
HUVEC
HUVEC
HUVEC
Rho-0
Rho-0
Rho-0
1000
500
200
75
(bp)
16S rRNA PCR product
SncmtRNA PCR product
Actin PCR product
