# Supplementary figures and images for: Regulation of nuclear transcription by mitochondrial RNA in endothelial cells
Source: eLife. 2024 Jan 22;13:e86204. doi: 10.7554/eLife.86204 (PMC10803041; doi:10.7554/eLife.86204)

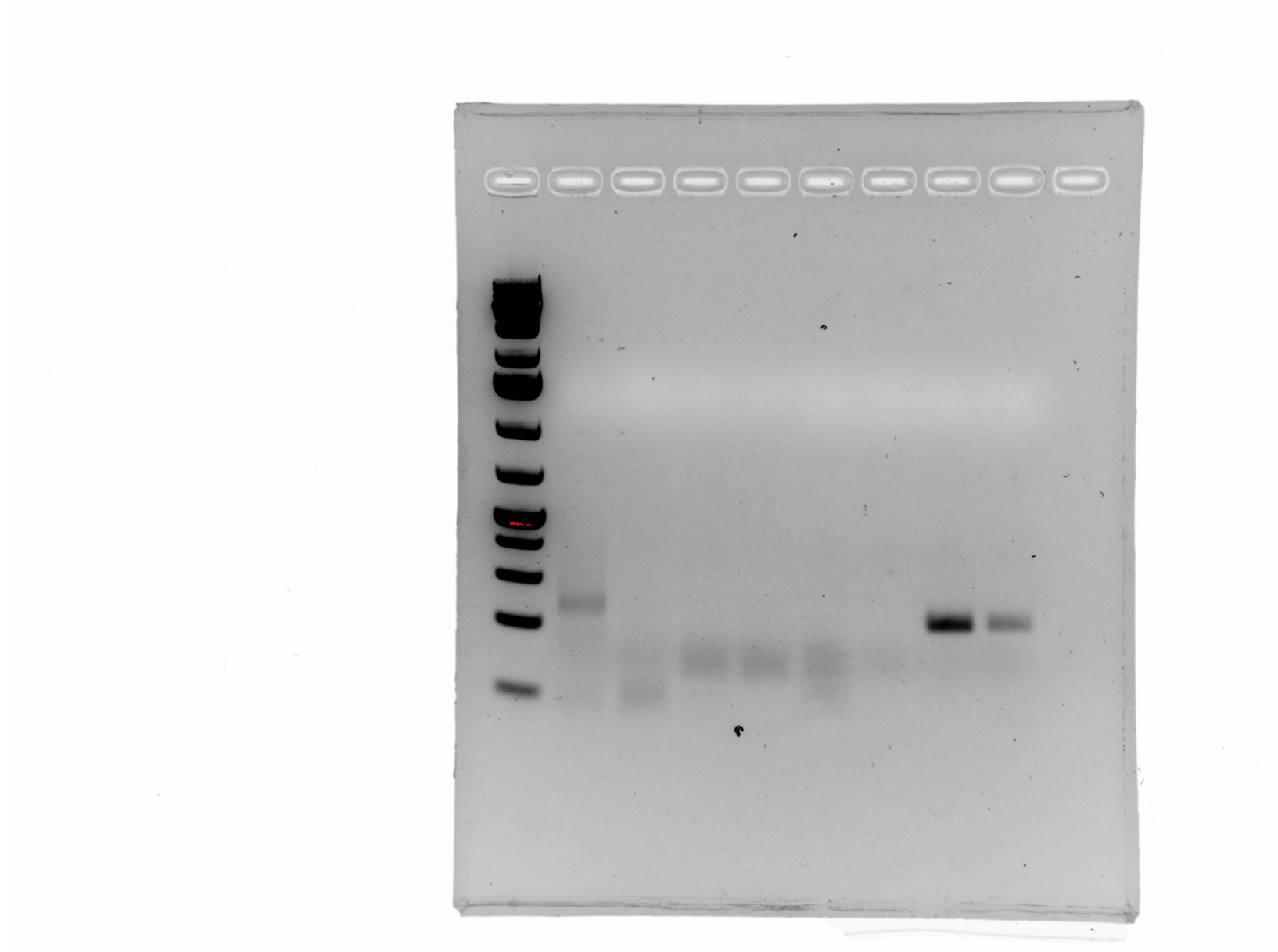

Supplement: Figure 2—source data 3. [file elife-86204-fig2-data3.zip › Figure 2 - source data 1.tif]

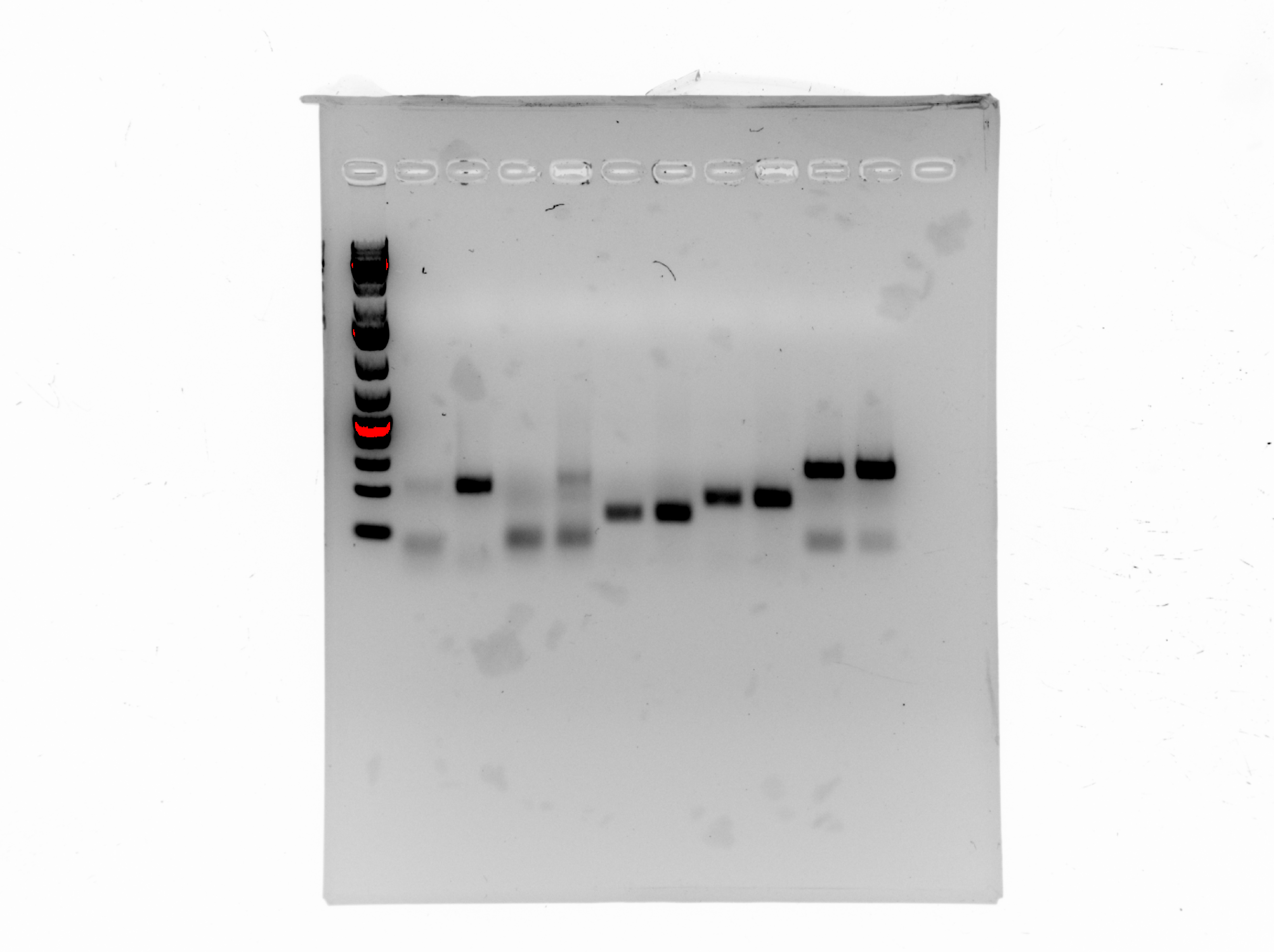

Supplement: Figure 2—source data 3. [file elife-86204-fig2-data3.zip › Figure 2 - source data 2.tif]
